# NRAMP1 and VDR gene polymorphisms in susceptibility to pulmonary tuberculosis among Andhra Pradesh population in India: a case–control study

**DOI:** 10.1186/s12890-017-0431-5

**Published:** 2017-06-05

**Authors:** Rooth Vasantha Medapati, Sridevi Suvvari, Sudhakar Godi, Paddaiah Gangisetti

**Affiliations:** Department of Human Genetics, Andhra University, Visakhapatnam, India

**Keywords:** Tuberculosis, NRAMP1, 3′UTR, 274CT, VDR, Fok1, Taq1, Andhra Pradesh population of India

## Abstract

**Background:**

The aim of the present study was to evaluate the association of NRAMP1 -3′UTR, 274-CT,VDR- Fok1 VDR-Taq1 Polymorphisms with the risk of pulmonary tuberculosis.

**Method:**

A case –control study was conducted on Andhra Pradesh Population of India. Analysis of gene polymorphisms of NRAMP1 gene (3′UTR, 274CT) and VDR gene (Fok1 and Taq1) was done by using Polymerase chain reaction-restriction fragment length polymorphism (PCR-RFLP) in Tuberculosis (TB) patients and healthy controls. The obtained results were observed using 2% Agarose Gel electrophoresis and analysed statistically using Chi-square test and Odds Ratio.

**Results:**

Statistical significance was observed between the patients and the controls in the NRAMP1-3′UTR (*P* = 0.005; OR = 2.997; 95% CI = 1.019–8.813) and VDR-Taq1 (*P* < 0.001; OR = 0.140;95% C.I = 0.050–0.386) polymorphisms in Andhra Pradesh population. No statistical significance was observed between patients and controls of the same population in NRAMP1-274CT and VDR-Fok1 polymorphisms (*p* > 0.05).

**Conclusion:**

3′UTR-NRAMP1 gene and VDR-Taq1 gene Polymorphisms are statistically associated with the susceptibility of TB in Andhra Pradesh Population in India.

## Background

Tuberculosis (TB) is a chronic bacterial infection caused by *Mycobacterium tuberculosis* (M.tb). It is transmitted from one individual to other by direct inhalation of droplet nuclei formed through coughing, sneezing etc. It can affect any part of the human body mainly the lungs causing Pulmonary Tuberculosis (PTB). It is the major cause of mortality and morbidity globally. The infection is multifactorial, both environmental and host genetic factors acting as the influencing agents [1]. In 2015, the global estimation of TB was 10.4 million new cases out of which 56% were males, 34% were females, 10% were children and 11% of the new TB cases were HIV co-infected. India accounts for more than one fourth of the world’s TB cases and deaths [2].

Two billion are estimated to be latently infected with M.tb, with 10% of them reactivating to an active lifetime disease. The reason may be due to the interaction of both environmental and a host of pathogen factors [3]. Research on TB revealed that genetic factors play a major role in the progression of TB. Several genes are found to be associated with PTB, two of them being NRAMP1 Gene (Natural -Resistance-Associated Macrophage Protein 1) and VDR (Vitamin D Receptor) candidate gene.

NRAMP1 gene is officially known as SLC11A1 (Solute Carrier family 11 proton-coupled divalent metal ion transporter) membrane1. It is located on long arm of Chromosome 2 (2q35) and exon count 16. NRAMP1 activates microbicidal responses in the infected macrophage hence it is important in the early innate response to mycobacterial infection. Iron is an essential mycobacterial nutrient. It is also required by the cell to generate reactive oxygen and nitrogen intermediates. NRAMP1 may control intracellular microbial replication by actively removing iron or other divalent cations from the phagosomal space [4, 5]. It encodes a multipass membrane protein. The protein functions as a divalent transition metal (Iron and Manganese) transporter involved in iron metabolism and host resistance to certain pathogens. Mutations in this gene are associated with susceptibility to infectious diseases and inflammatory diseases such as TB, Leprosy, Rheumatoid Arthritis and Crohn’s disease.

It was found that the mutant TGTG allele produce less amount of normal NRAMP1 protein than TGTG+ allele. The poly-A tail nucleotide length of mRNA in eukaryotes also consists of NRAMP1 mRNA. The poly-A tail gets degraded gradually by cytosolic nuclease when the eukaryotic mRNA enters the cytosol. As the poly-A tail gets completely destroyed the cytosol nuclease will then start to degrade the coding sequences of the tailless mRNA, thereby making the protein production to stop. The 3′UTR region has certain sequences needed to produce a normal length poly -A tail. It is an AATAA sequence followed by 23–24 nucleotides with a GT rich region. A loss of TGTG sequence within the GT rich region disturbs the normal poly- A tail formation [6, 7]. The TGTG del allele produces a short poly -A tail, hence its mRNA degrades earlier which results in less amount of NRAMP1 protein production when compared to TGTG+ allele which has a long poly - A tail.

VDR gene is located on the long arm of Chromosome 12 (12q13.11). Vitamin D can be obtained directly from diet and it can also be synthesised by the human body on exposure to sunlight. But this form of vitamin D is biologically inactive. This inactive form of vitamin D is delivered to the liver where it undergoes the first hydroxylation to become 25- hydroxyvitamin D. From the liver the 25-hydroxyvitamin D travels to the kidney where it undergoes another hydroxylation to become 1α- 25-dihydroxy vitamin D. This biologically active form of vitamin D affects the immune function. It regulates the activity of defense immune system [8–11]. In TB infection, vitamin D binds to vitamin D receptor in macrophages. The binding of vitamin D to VDR in macrophages helps in synthesis of cathelicidin in the lysosome of macrophages. This antimicrobial peptide (cathelicidin) plays a major role in innate immune defense against rapid spread of bacterial infection [12].

Thus cathelicidin restricts the growth of M.tb in Macrophages [12]. Polymorphism in VDR may lead to predisposition to PTB. Some of the Single Nucleotide Polymorphisms (SNPs) in VDR gene are Fok1, Taq1, Bsm1 and Apa1 [13]. The present study is focussed to assess the role of polymorphisms of NRAMP1 gene-3′UTR and 274C/T and VDR gene polymorphisms of Fok1 and Taq1 on PTB susceptibility by conducting a case–control study in Andhra Pradesh population in India. The variability between frequencies of the gene polymorphisms help in assessing the risk of TB in the Indian population.

## Methods

A case–control study was conducted on individuals aged between 11 and 70 years using Acid Fast Bacillus smear Test on early morning sputum for two consecutive days, chest X-ray of patients who were diagnosed with positive tuberculin skin test and clinically diagnosed as infected with TB at Revised National TB Control Program (RNTCP) centres of Chest and TB Hospital, Visakhapatnam were taken as PTB cases. PTB patients co-infected with Human Immunodeficiency Virus (HIV) were excluded in the study. Healthy individuals belonging to the same geographical area with no history of previous TB, though they were exposed to TB causing environment, and were fulfilling the eligible criteria like age, and gender were taken as controls. Informed consent was taken from all the participants before collecting the blood sample. The study was approved by the Institutional Ethics Committee for Research on Human Volunteers, Andhra University, Visakhapatnam, Andhra Pradesh, India. 2–5 ml of venous blood was drawn into ethylenediaminetetraacetic acid (EDTA) lined test tubes and were taken to the University Laboratory at−20°c, for Deoxyribo Nucleic Acid (DNA) extraction.

### Genotyping

Two SNPs of NRAMP1 gene (3′UTR and 274 C/T) and two SNPs in VDR gene (Fok1 and Taq1) which were found to be associated with pulmonary tuberculosis were selected for the study.

Polymorphisms in a gene can be detected by Polymerase Chain reaction (PCR) and Restriction Fragment Length Polymorphism (RFLP). The primers used for the amplification of NRAMP1 gene and VDR gene SNPs were represented in Table 1. Genomic DNA was extracted from venous blood and quantitative analysis was done using spectrophotometer.

**Table 1:** NRAMP1 and VDR Polymorphisms Sites and sequence variants

Gene	Primer sequence 5′ to 3′	Nucleotide and amino acid change	Genotypes	Fragment (bp)	Reference
NRAMP1 -3′UTR	For- GCA TCT CCC CAA TTC ATG GT	TGTG deletion in the 3′UTR of exon 15	TGTG++/TGTG++	244,	[36]
			TGTG+/TGTGdel	244,211	
	Rev- AAC TGT CCC ACT CTA TCC TG		TGTGdel/TGTGdel	211	
NRAMP1-274C/T	For-TGC CAC CAT CCC TAT ACC CAG	Silent nucleotide substitution in codon 66 (Phe) in exon 3	C/C	102	[36]
			C/T	102,167	
	Rev- TCT CGA AAG TGT CCC ACT CAG		T/T	167	
VDR-Fokl	For- AGC TGG CCC TG CAC TGA CTC TGC TCT	T → C transition in exon 2 initiation codon	C/C-FF	267	[14]
			T/C-Ff	267,197,70	
	Rev- ATG GAA ACA CCT TGC TTC TTC TCC CTG		T/T-ff	197,70	
VDR-Taql	For- CAG AGC ATG GAC AGG GAG C	C → T Transition in codon 352 at the 3′ end of exon 9	T/T-TT	455	[14]
			T/C-Tt	455,290,165	
	Rev- AGG AGA GGC AGC GGT ACT G		C/C-tt	290,165	

Note: For the NRAMP1 gene, individuals were scored as 3′UTR-TGTG+/+ wild homozygous, 3′UTR-TGTG+/del heterozygous, 3′UTR-TGTGdel/del mutant homozygous. ‘+’ represents presence of TGTG; ‘del’ represents absence of these four bases. For the NRAMP1-274C/T, VDR-Fokl,VDR-Taq1,individuals were scored as 274C/T-C/C, Fok1-FF,Taq1-TT wild homozygous, 274C/T-C/T,Fokl-Ff,Taq1-Tt heterozygous, 274C/T-T/T,Fokl-ff,Taq1-tt mutant homozygous respectively

Qualitative analysis is done using gel electrophoresis. Fok1 enzyme from New England Biolabs Inc (NEB), and Taq1 and Mnll from Thermo Scientific, India, were used to genotype polymorphisms in NRAMP1 and VDR genes. The PCR reaction mixture contained 12.7 μl of sterile water, 2.0 μl of 10× PCR buffer, 0.8 μl of deoxyNucleoside TriPhosphate (dNTPS), 0.5 μl of Taq DNA polymerase, 2 μl of Isolated DNA, 1 μl of forward primer, 1 μl of reverse primer.

The NRAMP1 3′UTR region is the 3′ untranslated region which starts from 55 nt 3′ to the last codon in the exon 15. In this region TGTG deletion was found. The PCR products consist of about 244 base pairs (bp) which are formed by the primer. The NRAMP1 274 C/T polymorphism correspond to a silent nucleotide substitution in codon 66 (phe) in exon 3 of the gene. The PCR product is about 167 bp. NRAMP1 274 C/T allele may be linked with low innate macrophage function. Allele specific oligonucleotide primers Fok1 and Taq1 were taken into study based on published VDR sequences [14]. The Fok1 polymorphism is found in the coding region of the VDR gene and is caused by T → C transition in exon 2 initiation codon. Primers for Fok1 amplified a sequence of about 267 bp in length. The Taq1 polymorphism is observed in the coding region of the VDR gene and is caused by a single base change C to T in codon 352 at the 3′end of VDR gene. Primers for Taq1 amplified a sequence of about 455 bp in length.

DNA amplification was performed in a Thermal Cycler (Bio-Rad) in 0.2 ml PCR tubes programmed as initial denaturation for 5 min at 95 °C (3′UTR,274 C/T,VDR-Taq1), and 96 °C (VDR-Fok1), followed by 35 cycles as denaturation for 30 sec at 95 °C, annealing for 30 sec at 52 °C (3′UTR), 56 °C (274 C/T), 60 °C (VDR-Fok1), 58 °C (VDR-Taq1) and extension for 30 sec at 72 °C. The final extension step was carried out for 10 min at 72 °C and the reactions were kept at 4 °C [15].

The PCR products were purified and restriction digestion was performed with Fok1 (3′UTR, VDR-Fok1), MnII (274 C/T), at 37 °C for 2 hours, Taq1 (VDR-Taq1) 65 °C for 1 h. The resultant products were separated on 2% agarose gel electrophoresis. The obtained bands for NRAMP1 3′UTR showed normal allele TGTG+ at 211 bp, TGTG+/+ (wild homozygous) at 211 bp, TGTG+/del (heterozygous) at 211 bp and 244 bp, TGTGdel/del (mutant homozygous) at 244 bp. Bands for NRAMP1-274 C/T showed C/C (wild homozygous) at 102 bp, C/T (heterozygous) at 102 bp and 167 bp, T/T (mutant homozygous) at 167 bp. The amplified sequence by the restriction enzyme Fok1 for VDR-Fok1 was named as (F/f) according to the base change (T/C) that was identified. The obtained bands shows F/F-C/C (wild homozygous) at 267 bp, F/f-T/C (heterozygous) at 267 bp and 197 bp, ff-T/T (mutant homozygous) at 197 bp and that of VDR-Taq1 with restriction enzyme Taq1 is named as (T/t) according to the base change (T/C) that was observed. The obtained bands show T/T-TT (wild homozygous) at 455 bp, T/C-Tt (heterozygous) at 455 bp, 290 bp and 165 bp, C/C- tt (mutant homozygous) at 290 bp and 165 bp (Table 1).

### Statistical analysis

Microsoft excel, SPSS version13.0 Statistical package and medcalc statistical software were used to perform the data analysis. *P* < 0.05 was used as the approach of statistical significance. Chi-square test was performed to calculate the genotype frequencies between the patients and controls [16]. The genotype frequencies and the allele frequencies in a population remain constant from generation to generation, if no evolutionary factors like mutation occur. But due to the presence of mutations these frequencies deviate from the Hardy-Weinberg Equilibrium (HWE). Odds Ratio (OR) and 95% confidence interval (C.I) were calculated to assess the degree of association between these SNPs and Tuberculosis. The association between SNPs and the risk of PTB was represented in three genetic models (wild homozygous, heterozygous, mutant homozygous).

## Results

The association between NRAMP1 3′UTR, NRAMP1 274C/T, VDR-Fok1, VDR-Taq1 and PTB risk are listed in Table 2. It was observed that for NRAMP1 3′UTR, in the heterozygous genotype TGTG+/del (OR = 2.997; 95%C.I = 1.019–8.813; *P* = 0.005) increased PTB risk by 2.99 fold and for VDR Taq-1, in the heterozygous genotype Tt (OR = 0.140; 95%C.I = 0.050–0.386; *P* < 0.001), the heterozygosity among the controls is most probably protecting them against TB. The mutations have affected the Hardy Weinberg Equilibrium (*P* < 0.05) with regard to NRAMP1 3′UTR and VDR-Taq1, but not with regard to mutations in NRAMP1 274C/T and VDR-Fok1, though they were mutations but not significant enough to disturb the equilibrium. The frequencies of 3′UTR, 274C/T, Fok1 and Taq1 SNPs in patients with TB were 20.9%, 26.1%, 94.4%, 69.6% respectively. The TGTG del, t, f, t allele frequencies of 3′UTR, 274C/T,Fok1, Taq1 SNPs in patients with TB were 13.7%,13.0%,51.7%,39.6% respectively (Table 2).

**Table 2:** Association between the SNPs genotypes of NRAMP1, VDR and the risk of Pulmonary Tuberculosis

Gene	polymorphism	type	patients		controls		Χ^2^	Odds ratio	95% C.I	P
			N	%	N	%				
NRAMP1-3′UTR	TGTG++	Wild homozygous	72	79.1	83	94.3	10.289	1		0.005*
	TGTG+/del	Heterozgous	13	14.3	5	5.7		2.997	1.019–8.813	
	TGTGdel/TGTGdel	Mutant homozygous	6	6.6	0	0.0		14.972	0.829–270.38	
	Alleles									
	TGTG		157	86.3	171	97.2	13.835	1		<0.001*
	TGTGdel		25	13.7	5	2.8		5.445	2.035–14.573	
NRAMP1-274C/T	C/C	Wild homozygous	68	73.9	69	83.1	5.559	1		0.062
	C/T	Heterozygous	24	26.1	12	14.5		2.029	0.940–4.381	
	T/T	Mutant homozygous	0	0.0	2	2.4		0.202	0.009–4.304	
	Alleles									
	C		160	87.0	150	90.4	1.000	1		0.317
	T		24	13.0	16	9.6		1.4063	0.719–2.750	
VDR-Fok1	FF	Wild homozygous	5	5.6	12	14.5	4.543	1		0.103
	Ff	heterozygous	76	85.4	61	73.5		2.9902	0.999–8.950	
	ff	Mutant homozygous	8	9.0	10	12.0		1.920	0.474–7.766	
	Alleles									
	F		86	48.3	85	51.2	0.287			0.592
	f		92	51.7	81	48.8		1.122	0.735–1.713	
VDR-Taq1	TT	Wild homozygous	27	29.3	5	5.9	17.719	1		<0.001*
	Tt	heterozygous	56	60.9	74	87.1		0.140	0.050–0.386	
	tt	Mutant homozygous	8	8.7	6	7.1		0.246	0.059–1.026	
	alleles									
	T		110	60.4	84	49.4	4.321	1		0.030*
	t		72	39.6	86	50.6		0.639	0.418–0.975	
*P value significant @5% level (*P* < 0.05)										

Note: Genotype and allele frequencies were compared between the patients and controls by χ2 test. *N* = sample size, 95% C.I = 95% confidence interval. * = *p* value <0.05 indicates statistical significance

The data showed that ‘TGTGdel’ allele frequencies of NRAMP1-3′UTR in patient group was more than in the control group indicating significant difference between the two groups. No significant difference in ‘t’ allele of NRAMP1-274C/T and ‘f’ allele of VDR-Fok1 was observed between the TB patients and the controls [(*P* = 0.317,0.103) (Table 2)].

Our results showed that the polymorphisms of the NRAMP1 gene (3′UTR) is significantly associated with PTB and may be risk factor for the development of TB and polymorphisms of VDR gene (Taq1) as a protective factor in Andhra Pradesh population of India. Our results also indicated that the polymorphism in the 274C/T of NRAMP1 gene and Fok1 of the VDR gene are not associated with TB in this population (*P* = 0.062 and 0.103) (Table 2).

## Discussion

The association between PTB and NRAMP1, VDR genes was studied for the first time in Andhra Pradesh population of India. The genic association differs with different ethnicities. The small number of the study sample may also obfuscate the results. The interactions between different genes and environmental factors play an important role in the action of VDR gene. The metabolism of vitamin D causes the activation of macrophage which restricts the intracellular growth of M.tb. [17–19]. Deficiency of vitamin D was common in TB patients and should be associated with TB susceptibility. The obtained results were compared with the results of some previously published studies with other population. It was observed that 3′UTR, INT4, D543N and 5′(GT)n) polymorphisms of NRAMP1 were significantly associated with TB in West Africans [1]. Significant association of 3′UTR polymorphisms with TB was revealed in Koreans and Chinese Han population [20–22]. Molecular Study on Chinese Kazakh population found significant association of 3′UTR with TB [14]. Furthermore associative studies conducted on NRAMP1-3′UTR polymorphisms and the risk of TB were found to give significant results in Japanese and South African population [23, 24].

However, no association was found between 3′UTR polymorphism of NRAMP1 gene and TB in Taiwan population [25]. Similar studies conducted on Thai, Moroccan, Dane and Brazilian population gave results which did not show significance between the risk of TB and NRAMP1 gene [26–29].

In our study, it was found that 3′UTR polymorphism have a high risk for TB in Andhra Pradesh Indian populations. OR = 2.997, (95% C.I = 1.019–8.813) *P* = 0.005 (Table 2).

The study also showed that 29.3% of patients were homozygous to ‘TT’ genotype and ‘tt’ of VDR-Taq1 gene were higher in the patient group than in the control group. The chi-square value (17.719) (Table 2) revealed significant variation in the distribution of the three genotypes TT, Tt, tt between the patients and controls in the present study. There appears a significant association of Taq1 SNP of VDR gene with TB indicating protection from TB in the present study population.

In this study no significant difference of Fok1 Ff, and ff genotype frequencies was observed between TB patients and controls (*P* = 0.103) (Table 2). Different studies by various researchers on association of TB with NRAMP1 AND VDR polymorphisms gave different results due to ethnic differences [30]. The interaction varies from one population to another population. This heterogeneity is also contributed by several influences of vitamin D3 on the transcription process of various endocrine pathways [19, 31]. Polymorphisms of VDR gene are found to be associated with resistance to TB in Gambian population [11]. Significant association was found between Fok1 and Taq1 Polymorphism of VDR gene in Gujarati Asians living in West London [18, 32]. But no association was observed between TB patients in Tuvinian population and Fok1 Polymorphism of VDR gene [33]. Additionally studies done on VDR gene and PTB in Cambodian and Tanzanian population also proved as non-significant [34, 35]. No association was noticed between the disease and Fok1 polymorphisms in Chinese Kazakh and Chinese Han population [14, 22].

## Conclusion

The present study reports that the polymorphism of NRAMP1 gene (3′UTR) and VDR gene Taq1 are significantly associated with TB in Andhra Pradesh population of India. But 274 C/T of NRAMP1 and Fok1 of VDR genes are not associated with TB in the present study population. The observations of the present study support the fact that gene polymorphisms are multifactorial in nature being influenced by ethnic, geographical and environmental factors.

NRAMP1 genotype identification has potential contribution in the clinical management of TB. But, further studies on the host loci, immune markers and disease severity should be taken into account to assess the importance of the genetic variants in different populations and also to understand the key factors in host immunity to TB. Further analysis on whether NRAMP1 and VDR gene variants directly affect NRAMP1 and VDR gene functions or whether other functional polymorphisms of these two genes exist is necessary to help in public health control measures of TB.

## Abbreviations

bp: Base pair
C.I: Confidence interval
Df: Degree of freedom
DNA: Deoxyribo nucleic acid
dNTPS: DeoxyNucleoside TriPhosphate
EDTA: Ethylenediaminetetraacetic acid
HIV: Human immunodeficiency virus
M.tb: Mycobacterium tuberculosis
NEB: New England Biolabs
NRAMP1: Natural –resistance-associated macrophage protein 1
OR: Odds ratio
PCR: Polymerase chain reaction
PTB: Pulmonary tuberculosis
RFLP: Restriction fragment length polymorphism
RNTCP: Revised national TB control program
SLC11A1: (Solute carrier family 11 proton-coupled divalent metal ion transporter) membrane1
SNP: Single nucleotide polymorphism
TB: Tuberculosis
VDR: Vitamin D receptor
